# The Influence of Transaction Process With Doctors on Patient Satisfaction, Self-Rating Anxiety and Self-Efficacy Among International Students in China

**DOI:** 10.3389/fpubh.2021.737278

**Published:** 2021-09-23

**Authors:** Janelle Julien, Xuemei Wang, Han Meng, Zhou Qian, Dan Wang, Xinping Zhang

**Affiliations:** ^1^School of Medicine and Health Management, Tongji Medical College of Huazhong University of Science and Technology, Wuhan, China; ^2^School of Management, Hubei University of Chinese Medicine, Wuhan, China

**Keywords:** international student, patient satisfaction, theory of goal attainment, transaction process, self-efficacy, self-rating anxiety

## Abstract

**Objectives:** To investigate the communication mechanism between international students and Chinese physicians by evaluating the influence of the transaction process on patient satisfaction, self-rating anxiety and self-efficacy.

**Methods:** A cross-sectional survey was conducted among international students living in Central, Northern and Eastern China; enrolled at Chinese universities and experienced outpatient and inpatient healthcare services. Guided by the elements of King's transaction process: IR, Initiating and Responding; IP, Identifying Problems; MGS, Mutual Goal Setting; and EM, Exploring means and agreeing on means to achieve goals. We used spearman correlation analysis to calculate the correlation of the variables: patient satisfaction, self-efficacy, transaction process, IR, IP, MGS, and EM and regression analysis to measure the influence of transaction process on patient satisfaction, self-rating anxiety and self-efficacy.

**Results:** Four hundred and four (404) participants were investigated for this study. The results of correlation analysis showed that there was a significant positive correlation among patient satisfaction, self-efficacy, transaction process, IR, IP, MGS, and EM (*p* < 0.05). Regression analysis showed that the higher scores of IR (β = 0.176, *p* = 0.003) and MGS (β = 0.249, *p* = 0.002) was associated with the higher score of patient satisfaction; the higher the score of IR and IP was associated with the higher self-efficacy score (β = 0.148,0.225; *p* = 0.016,0.001); and higher the MGS score was associated the lower the self-rating anxiety (β = −0.220, *p* = 0.022).

**Conclusion:** The influence of transaction process on patient satisfaction, self-rating anxiety and self-efficacy between Chinese physicians and international students (Patients) was established. Findings support the urgent implementation of tools at healthcare facilities to improve the communication between Chinese doctors and international students, therefore improving patient satisfaction and self-efficacy, and reducing anxiety.

## Introduction

The global flow of international students is consistently increasing (1). A report indicated that 492, 185 international students are pursuing higher education in mainland China and had an increase of 3,013 in 2018 (2). Most of these students come from Asian and African countries, (2) and experience significant difficulties from assimilating into new customs to access healthcare under the unique cultural practices in host country (3, 4). One study revealed that around 71% of students agreed that language was a barrier to healthcare access and interpreter services should be available (5). Language barriers can hinder communication and reduce patient satisfaction. (6). Studies have also shown that poorer health outcomes with patients is positively associated to their experience language barriers (7, 8). A study highlighted that English speakers are less likely to experience adverse events related to communication errors in USA (9).

Many studies on international students' communication in health care service showed that linguistic discordance was a prevailing challenge in their utilization of healthcare services (10, 11). One study showed that even with accessible online communication tools, students were faced with communication difficulties (12). The main findings indicate discrepancies with host language, which presents a gap for in-depth research in the actual interaction between doctor and international student.

This problem can result in misunderstandings and potentially reduce the quality of healthcare service (13).

Effective communication between doctor-patient is essential for optimum healthcare delivery but is challenging (14, 15). Recently, communication has been given significant attention in attaining high quality healthcare services (16, 17). King's Theory of Goal Attainment (TGA) was developed and used to promote communication between health workers and patients (18). One aspect of King's theory; the transaction process, emphasized the importance of setting health goals with the patient and pursue the necessary guidelines to accomplish those goals. In the hospital environment, health workers interact with patients, with the intent to form mutually selected goals, when this mode of communication is established, the goals are likely to be attained (18). Communication is an invaluable facet in attaining high quality healthcare services. Miscommunication in doctor-patient communication is a growing problem (19) and further attention is needed, with special focus on the communication level with international students.

Many studies have showed that communication skills are a major factor in patient outcomes and nurturing patient satisfaction (20, 21). It is a also common belief that physicians talking to patients reduces patient anxiety and increases patient satisfaction (22–24).

Another concern linked with communication is self-efficacy. Self-efficacy is concerned with people's beliefs in their ability to influence events that affect their lives (25). *Many* studies have showed that communication skills are a major factor in patient self-efficacy, (26, 27) but there is no report on the relationship between doctor-international students communication with patient satisfaction, anxiety and self-efficacy which is valuable for international students.

Creating a link between international student's communication with Chinese physicians is vital as students are from diverse backgrounds and may encounter difficulties in the hospital environment. *The Problems of this study are b*ased on the gap of limited studies:

What is the communication level between doctors and international students based on King's transaction process?

Does the communication level between doctors and international students influence patient satisfaction, anxiety and self-efficacy?

## Materials and Methods

### Participants

The respondents resided in Northern, Eastern and Central China. A sample size of 384 was deemed necessary to conduct the survey with a population size of 492,185, confidence level of 95% and margin of error at 5%, using a sample size calculator [https://www.surveymonkey.com/mp/sample-size-calculator/]. Ethics approval was obtained from the University before the questionnaire survey was disseminated. Participants were contacted through online social networks, namely, WeChat and WhatsApp.

### Materials

It mainly covered the measures of the transaction process and the outcomes. The primary outcome measures were the levels of patient satisfaction, self-rating anxiety and self-efficacy. Patient satisfaction refers to the “extent to which patients are happy with their healthcare,” (28) this extends to both inpatient and outpatient experiences; anxiety is a feeling of fear and uneasiness, (29) whilst consulting with Chinese physicians; and self-efficacy represents the belief that students are capable of being assertive in implementing approaches to achieve their healthcare goals despite difficulties (25). The secondary outcome measures were age, Chinese proficiency and residence of participants in China and whether these characteristics predicted the outcomes. Main study instruments are presented as follows.

#### The Transaction Process

In this study, the theory of goal attainment (TGA) is applied in the hospital context because it provides an understanding of the international student-physician mechanism and attaining patient satisfaction. King's transaction process was established to to achieve better health outcomes through communication with patients, setting goals, exploring means and agreeing on means to achieve goals and the framework was tested and validated in the pharmaceutical context (30). The elements are as presented as follows:

i. Initiating and responding: One party initiates an action, one party responds.
ii. Identifying problems: Through interaction, a problem is identified.
iii. Mutual goal setting: Feedback is expressed and a goal is mutually agreed upon.
iv. Exploring means and agreeing on means to achieve goals: The physician and international student explore means to achieve the goal and reach mutual agreement on means to achieve the goal.

Additionally, the relationships with the transaction process, patient satisfaction, self-rating anxiety and self-efficacy were explored.

This scale evaluates the King's transaction process in the healthcare context between international student patients and Chinese physicians. The theory postulates that the health professional and the patient interact to establish transactions. Goals are established and both parties work to accomplish them on a five-point scale ranging from (1) never to (5) always based on the quality of interaction between international student and Chinese physician. The initial scale, Adolescent Transaction Scale (ATS) was revised for use in the pharmaceutical care context, (30) then revised for medical care service for this study. The scale was created using the elements of King's theory and measured initiation and response, identification of problems, mutual goal setting and exploration of means and agreeing on means toward goals. After expert consultation, all items were retained and reworded to fit medical care service.

#### Patient Satisfaction

This scale aimed to evaluate international student's satisfaction on medical-service seeking experience in Chinese hospitals. Because language is a major challenge during the medical consultation between international student patients and Chinese physicians, thus an. additional item “The medical communication/consultation with physicians (especially on the aspect of language)” was added in item 7 on a five-point Likert scale ranging from (1) not satisfied at all to (5) very much satisfied.

#### Self-Rating Anxiety

This scale aimed to evaluate the anxiety level of international students seeking medical advice in hospitals in China. The Hamilton Anxiety Scale (HAM-A) is a reliable and validated scale. The scale was developed and used to measure the severity of anxiety symptoms during medical consultation with Chinese physicians. It includes 14 items, each measuring a medical symptom (31). Participants were requested to indicate the presence of these symptoms by answering (1) not present to (5) severe.

#### Self-Efficacy

This scale aimed to evaluate the self-confidence of international students when seeking medical advice at hospitals in China, because seeking medical advice in Chinese hospitals is a challenge for international students. General self-efficacy (GSE) is the belief in one's ability to embrace novel challenges and implement strategies to tackle tasks (32). The GSE was modified to fit the hospital setting. A response of (1) none of the time to (4) most or all of the time, to indicate the confidence level of finding hospital to understanding the physician and coping with other medical staff was measured.

### Data Collection

Potential participants were invited to participate in this online survey using Google Forms. The survey consisted of multiple choices questions regarding demographic characteristics, the transaction process, patient satisfaction, self-rating anxiety and self-efficacy. Full instructions were given at the beginning of the survey and participants were given the surety of confidentiality.

The transaction process scale yielded Cronbach's alpha of 0.94. Survey questionnaires had content validity as each section contained vital components inclusive of patient care, promptness of care, assertiveness in seeking help and psychological state prior to visiting hospital.

### Data Analysis

Descriptive analysis was done on the demographic characteristics of respondents. Also, the mean and standard deviation of the elements of the transaction process, patient satisfaction, self-rating anxiety and self-efficacy was conducted. For the correlational analysis; an assessment of a possible relationship among the elements of the transaction process with patient satisfaction, self-rating anxiety and self-efficacy using spearman correlation coefficient was done. Regression analysis; after dividing the elements of the transaction process into independent variables, demographic characteristics were used as control variables, whilst patient satisfaction, anxiety and self-efficacy were dependent variables. Multiple linear analysis was then conducted to predict the value of the independent variables (IR, IP, MGS and EM) on the dependent variables (patient satisfaction, self-rating anxiety and self-efficacy). The Statistical Package for Social Sciences for Windows (SPSS, version 20) was used to conduct the statistical analysis.

## Results

### The Demographic Characteristics of Students

Four hundred and four (404) students were investigated for this study. The demographic characteristics of students are presented in Table 1. Among the 404 students, 50.2% were female, and 49.8 were male. Most of the international students are from North America (45%), and most of them have been in China for 3–5 years (57.2%). And 33.7% of the participants “do not speak Chinese” 0.70% of participants believe that “language barrier is a significant concern in seeking healthcare in China” (Table 1).

**Table 1:** Demographic Characteristics of Participants (*N* = 404).

**Characteristic**	***N***	**%**
**Gender**		
Male	201	49.8
Female	203	50.2
Age (years)	29.33 ± 5.15	
**Country of origin**		
North America	182	45.0
South America	45	11.1
Europe and Australia	55	13.6
Africa	96	23.8
Asia	26	6.4
**Length of residence in china**		
<1 year	25	6.2
1–2 years	113	28.0
3–5 years	213	52.7
More than 5 years	53	13.1
**Residence in china**		
Central area	197	48.8
Northern area	121	30
Eastern area	86	21.3
**Chinese level**		
Do not speak Chinese	136	33.7
Elementary	88	21.8
Intermediate	138	34.2
Advanced	42	10.2
**Accommodation in china**		
On campus	322	79.7
Off campus	82	20.3
**Have you ever sought health care services in China?**		
Yes	404	100
No		
**Health insurance: out of pocket**		
Yes	142	35.1
No	262	64.9
**Variables**	**Mean**	**SD**
Initiating and responding	2.94	0.68
Identifying problems	2.56	0.75
Mutual goal setting	2.53	0.73
Exploring means and agreeing on means to achieve goals	2.74	0.44
Transaction process	2.70	0.56
Patient satisfaction	2.90	1.01
Self-rating Anxiety	2.72	0.47
Self-efficacy	1.22	0.39

### Transaction Process and Outcomes

Transaction process consists of Initiating and Responding (IR), Identifying Problems (IP), Mutual Goal Setting (MGS), Exploring Means and Agreeing on Means to Achieve Goals (EM). IR (2.94 ± 0.68) scored the highest, followed by EM (2.74 ± 0.44), IP (2.56 ± 0.75), MGS (2.53 ± 0.73). The average value of total score of transaction process is (2.70 ± 0.56), the average value of total score of patient satisfaction is (2.90 ± 1.01), self-rating anxiety is (2.72 ± 0.47), and self-efficacy is (1.22 ± 0.39) (Table 1).

### Correlation Analysis of the Transaction Process With Patient Satisfaction, Self-Rating Anxiety and Self-Efficacy

The results of correlation analysis (Table 2) showed that there was a significant positive correlation between the variables of patient satisfaction, self-efficacy, transaction process, IR, IP, MGS, and EM (*p* < 0.05). And there was a significant negative correlation between self-rating anxiety and other variables such as patient satisfaction, self-efficacy, transaction process, IR, IP, MGS, and EM (*p* < 0.05).

**Table 2:** Spearman correlation analysis.

	**IR**	**IP**	**MGS**	**EM**	**Transaction process**	**Patient satisfaction**	**Self-rating anxiety**	**Self-efficacy**
IR	1							
IP	0.560	1						
MGS	0.531	0.722	1					
EM	0.645	0.743	0.800	1				
Transaction process	0.764	0.841	0.897	0.915	1			
Patient satisfaction	0.450	0.490	0.535	0.516	0.557	1		
Self-rating anxiety	−0.152	−0.317	−0.368	−0.327	−0.348	−0.265	1	
Self-efficacy	0.388	0.481	0.469	0.458	0.515	0.468	−0.298	1

*IR, Initiating and Responding; IP, Identifying Problems; MGS, Mutual Goal Setting; EM, Exploring Means and Agreeing on Means to Achieve Goals*.

### Regression Analysis of the Influence of Transaction Process on Patient Satisfaction, Self-Rating Anxiety and Self-Efficacy

IR, IP, MGS, EM were divided into independent variables, demographic characteristics were taken as control variables, and patient satisfaction was taken as dependent variables. Multiple linear regression analysis was conducted. The results showed that the higher the scores of IR (β = 0.176, *p* = 0.003) and MGS (β = 0.249, *p* = 0.002), the higher the satisfaction was; the central area of China is taken as the reference, while the northern area has a higher degree of satisfaction (β = 0.105, *p* = 0.017), compared with “Do not speak Chinese,” the satisfaction of “Elementary” (β = 0.118, *p* = 0.010), “Intermediate” (β = 0.189, *p* = 0.000) and “Advanced” (β = 0.128, *p* = 0.009) group was higher.

IR, IP, MGS, EM were divided into independent variables, demographic characteristics were taken as control variables, and Self-efficacy was taken as dependent variables. The multiple linear regression analysis showed that the regression model passed the F test (F = 9.157, *P* < 0.05), and IR score, IP score and language barrier had significant predictive effects on Self-efficacy. The higher the score of IR and IP, the higher the Self-efficacy score (std β = 0.148, 0.225; *p* = 0.016,0.001). Those international students who believe the language barrier is a significant concern in seeking healthcare in China had lower Self-efficacy scores (std β = −0.257, *p* = 0.004).

IR, IP, MGS, EM were divided into independent variables, demographic characteristics were taken as control variables, and self-rating anxiety was taken as dependent variables. The multiple linear regression analysis showed that the higher the MGS score, the lower the self-rating anxiety (β = −0.220, *p* = 0.022); female's self-rating anxiety was higher than that of male (β = 0.136, *p* = 0.006); Compared with the participants from North America, the self-rating anxiety of Asia was lower (β= −0.139, *p* = 0.009) (Table 3).

**Table 3:** Regression analysis of the influence of transaction process on patient satisfaction, Self-rating anxiety and self-efficacy.

**Variables**		**Patient satisfaction**			**Self-efficacy**			**Self-rating anxiety**		
		**β**	**Std β**	***P***	**β**	**Std β**	***P***	**β**	**Std β**	***P***
	Cons	0.481		0.366	1.711		0.000	1.573		0.000
IR		0.261	0.176	0.003	0.102	0.148	0.016	0.059	0.103	0.142
IP		0.116	0.086	0.217	0.160	0.255	0.001	0.067	0.129	0.129
MGS		0.344	0.249	0.002	0.037	0.057	0.499	−0.118	−0.220	0.022
EM		0.186	0.081	0.355	0.087	0.081	0.380	−0.172	−0.192	0.069
Gender	Male		R			R			R	
	Female	0.011	0.005	0.893	0.008	0.009	0.837	0.106	0.136	0.006
Age	Age	−0.012	−0.061	0.136	−0.001	−0.015	0.731	−0.002	−0.029	0.559
Country of origin	North America		R			R			R	
	South America	−0.099	−0.031	0.478	0.049	0.033	0.478	0.003	0.002	0.964
	Europe and Australia	−0.035	−0.012	0.780	0.004	0.003	0.952	−0.003	−0.003	0.961
	Africa	0.007	0.003	0.948	0.002	0.002	0.969	−0.128	−0.139	0.009
	Asia	−0.049	−0.012	0.773	0.151	0.079	0.075	−0.054	−0.034	0.501
Length of residence in china	<1 year		R			R			R	
	1–2 years	0.071	0.032	0.692	0.145	0.138	0.103	−0.113	−0.130	0.180
	3–5 years	0.098	0.049	0.569	0.113	0.120	0.186	−0.130	−0.166	0.108
	>5 years	0.021	0.007	0.918	0.086	0.062	0.393	−0.083	−0.072	0.383
Location in china	Central area		R			R			R	
	Northern area	0.034	0.016	0.722	0.002	0.002	0.968	0.018	0.021	0.688
	Eastern area	0.258	0.105	0.017	0.095	0.082	0.077	−0.098	−0.102	0.054
Chinese level	Do not speak Chinese		R			R			R	
	Elementary	0.288	0.118	0.010	0.082	0.072	0.138	0.097	0.102	0.064
	Intermediate	0.403	0.189	0.000	0.101	0.102	0.053	−0.015	−0.018	0.760
	Advanced	0.424	0.128	0.009	0.044	0.029	0.581	0.114	0.089	0.134
Accommodation in china	On campus		R			R			R	
	Off campus	0.163	0.065	0.115	0.043	0.037	0.402	0.028	0.029	0.565
Health insurance: out of pocket	Yes	−0,071	R			R			R	
	No		−0.034	0.416	−0.034	−0.034	0.437	0.006	0.008	0.875
Language barrier	No		R							
	Yes	−0.206	−0.094	0.262	−0.264	−0.257	0.004	0.082	0.096	0.342
	Maybe	0.019	0.008	0.924	−0.108	−0.099	0.264	0.007	0.007	0.942
F		12.463			9.157			3.052		
${\text{R}}_{\text{adjust}}^{2}$		0.385			0.308			0.101		

*R(reference group). β, beta; Std β, Standardized beta; P, p-value.

## Discussion

The elements of the King's transaction process, showed direct and indirect effects among patient-related results, specifically, patient satisfaction, anxiety and self-efficacy. The transaction process proved to be a valid theoretical framework of student (patient)–physician communication in a medical care context, and the positive correlation among variables transaction process, patient satisfaction and self-efficacy with empirical data.

Imogene King's descriptive study on nurse-patient interaction laid the foundation for concrete elements required for successful communication and transaction in the nurse practitioner-patient setting (33). Previous studies have demonstrated and validated King's transaction process using empirical data (30, 34). These studies were conducted with nurses and in the pharmaceutical context, respectively. However, no validated study has been conducted in the medical care service context with Chinese physicians and international students.

Regarding the elements of the transaction process, distinctive associations were shown in relation to the study outcomes. For instance, high initiating & responding and mutual goal setting directly influenced patient satisfaction. Many studies have outlined that patient satisfaction improves communication (35). Similarly, findings in one tele health research (36) showed the importance of communication in mutual goal setting. Communication was shown to be an important aspect in successful patient therapy. Patients showed a major interest in discussing their treatment with doctors (37).

The strongest positive correlation among the elements of the transaction process were mutual goal setting and exploring means and agreeing on means to achieve the goals. One study demonstrated that goal-setting was liked by both patient and doctor. Both elements indicated its importance as improving patient-centered care (38). The results of this study also indicated a positive association between the transaction process and patient satisfaction. Consistent with these findings, King's model was appropriate in improving patient satisfaction in a study on diabetic patients (37).

High initiating and responding and identifying problems were associated with high self-efficacy. It is noted that patient- focused communication, results in high levels of self-efficacy (39). In one Rwandan investigation, self-efficacy was linked to low confidence in attaining mental health care but increased communication capacities, resulted in improved assertiveness with hospital staff and the ability to seek healthcare services (40). High mutual goal setting was associated with low anxiety. It is important to note that the female respondents' anxiety was higher compared to their male counterparts. Also, international students from Asia anxiety was significantly lower compared to North American students. Studies have shown that people who experience common anxiety symptoms, delay medical services (41). Establishing rapport with physicians can diminish or reduce anxiety in patients.

Host language competence also showed a major difference with students who “do not speak Chinese” on the aspect of patient satisfaction. In one study on communication with foreign patients, it was proven that patients with limited French proficiency was given assistance after failed communication and transaction attempts (42). A lack of awareness of the impact language disparities has on quality healthcare services may lead to low patient satisfaction.

The regression analysis showed Chinese proficiency (elementary, intermediate and advanced Chinese) influenced patient satisfaction and, language barrier is associated with lower self-efficacy scores. Communication between international students and physicians is the prerequisite for transaction to occur. The central focus of King's goal attainment theory is that physicians work with patients to achieve mutually agreed upon health goals. International students proficient in the Chinese language showed higher satisfaction and self-efficacy. The main concerns about communication is on the aspect of language. The challenges faced by international students on linguistic diversity was explicit in this study. Poor communication on the aspect of language barriers, presents a problem for the transaction process. Each element in the process, requires familiarity in terms of language from initiating and responding to exploring means and agreeing on means to achieve goals. Measures to improve the communication mechanism between international student and Chinese physician, and further improve the transaction process can be explored further.

This study has the following practical implications. Methodologically, this study used online survey to garner information on the influence of transaction process on patient satisfaction, anxiety and self-efficacy in the Chinese hospital environment. This yielded pertinent information on the realities faced by international students when interacting with Chinese doctors. Practically, this study provides some insights into communication mechanism in host hospital environment. The theoretical contributions of the current study are multifold. Firstly, it adds contributions to the field of healthcare services as it relates to international students by investigating how communication influences patient satisfaction. This study also addresses the neglected link in research between international students and the transaction process. Secondly, a large part of this research on the association between elements of the transaction process and anxiety and self-efficacy. Because of the small sample size, the present findings are to be interpreted with some caution. However, it is noted that similar studies yielded consistent findings. For doctors and international students, the findings show that the transaction process in the hospital setting is key for the satisfaction of international students.

The findings of this study are centered on encouraging hospitals to improve their service delivery. Optimistically, this study will play a vital role in the literature of the transaction process on patient satisfaction. Moreover, hospital administration should offer critical attention to international students' experiences and interaction within the hospital.

King's theory of goal attainment showed its appropriateness as a guide in providing health services to patients. The research projected the importance of communication, transaction, satisfaction, anxiety and confidence in the interaction between doctors and international student. However, in this study the results have implications for further research. Firstly, the development of a tool to improve the healthcare services between doctors and international students. Secondly, the current study is limited to international students in Central, Northern and Eastern China. Future studies could be conducted in other parts of China. Future studies are encouraged to examine or gain insights on the doctor's experiences with international students.

## Conclusion

According to King's TGA, effective paths between transaction process in the international student-physician relationship and patient-related outcomes were established. The problems faced by international students when visiting hospitals in China is evident. The positive association between transaction process and patient satisfaction and self-efficacy was established based on regression analysis. Identifying problems and language barrier were predictors for low self-efficacy and high mutual goal setting was associated with low anxiety. Most importantly, countermeasures are addressed to develop policies and procedures to improve linguistic and culture related care, to aid in the facilitation of the transaction process. Understanding the intricacies of King's TGA serves to enhance healthcare quality and improve health outcomes.

## Data Availability Statement

The raw data supporting the conclusions of this article will be made available by the authors, without undue reservation.

## Ethics Statement

The studies involving human participants were reviewed and approved by The Ethics Committee of the Tongji Medical College of Huazhong University of Science and Technology. The patients/participants provided their written informed consent to participate in this study.

## Author Contributions

XZ, DW, JJ, and ZQ helped design the study and drafted the initial manuscript. JJ, XW, and HM performed the data collection and initial analyses. JJ, XW, ZQ, HM, and DW reviewed and revised the manuscript. XZ helped revise the manuscript. All authors read and approved the final manuscript as submitted.

## Funding

This study was supported by the National Natural Science Fund Program, Theorizing Pharmacist-Patient Communication Model and Mechanism Based on King's Theory of Goal Attainment (Grant Number G040602, 2017) and the National Social Science Fund, Research on the Development Strategy of Advancing Health in China Based on National Health Coverage (Grant Number 15ZDC037, 2015).

## Conflict of Interest

The authors declare that the research was conducted in the absence of any commercial or financial relationships that could be construed as a potential conflict of interest.

## Publisher's Note

All claims expressed in this article are solely those of the authors and do not necessarily represent those of their affiliated organizations, or those of the publisher, the editors and the reviewers. Any product that may be evaluated in this article, or claim that may be made by its manufacturer, is not guaranteed or endorsed by the publisher.

## Abbreviations

TGA: Theory of Goal Attainment
GSE: General self-efficacy
HAM: A Hamilton Anxiety Scale
IR: Initiating and Responding
IP: Identifying Problems
MGS: Mutual Goal Setting
EM: Exploring means and agreeing on means to achieve goals.
